# Functional Restoration of Exhausted CD8 T Cells in Chronic HIV-1 Infection by Targeting Mitochondrial Dysfunction

**DOI:** 10.3389/fimmu.2022.908697

**Published:** 2022-07-05

**Authors:** Aljawharah Alrubayyi, Elia Moreno-Cubero, Dan Hameiri-Bowen, Rebecca Matthews, Sarah Rowland-Jones, Anna Schurich, Dimitra Peppa

**Affiliations:** ^1^Nuffield Department of Medicine, University of Oxford, Oxford, United Kingdom; ^2^Division of Infection and Immunity, University College London, London, United Kingdom; ^3^Centre for Sexual Health and HIV Research, University College London (UCL), London, United Kingdom; ^4^School of Immunology and Microbial Sciences, King’s College London, London, United Kingdom; ^5^Mortimer Market Centre, Department of HIV, Central and North West London NHS Foundation Trust, London, United Kingdom

**Keywords:** CD8 T cell exhaustion, HIV-1, CMV, immunometabolism, mitochondria, oxidative phosphorylation

## Abstract

CD8 T cell exhaustion is a hallmark of HIV-1 infection, characterized by phenotypic and functional CD8 T cell abnormalities that persist despite years of effective antiretroviral treatment (ART). More recently, the importance of cellular metabolism in shaping T cell antiviral function has emerged as a crucial aspect of immunotherapeutics aimed at re-invigorating exhausted CD8 T cells but remains under-investigated in HIV-1 infection. To gain a better insight into this process and identify new targets for effective CD8 T cell restoration we examined the metabolic profile of exhausted CD8 T cells in HIV-1 infection. We show that relative to HIV-1 elite controllers (EC) and HIV-1 seronegative donors, CD8 T cells from HIV-1 viraemic individuals are skewed toward a PD-1^hi^EOMES^hi^T-bet^low^TIGIT^+^ phenotype that is maintained during ART. This exhausted signature is enriched in HIV-specific CD8 T cells, compared to CMV-specific CD8 T cell populations, and further delineated by higher expression of the glucose transporter, Glut-1, impaired mitochondrial function and biogenesis, reflecting underlying metabolic defects. A notable improvement in antiviral HIV-specific CD8 T cell function was elicited *via* mitochondrial antioxidant treatment in combination with pharmacological modulation of mitochondrial dynamics and IL-15 treatment. These findings identify mitochondria as promising targets for combined reconstitution therapies in HIV-1 infection.

## Introduction

Generalized immune activation leading to T cell dysfunction and progressive exhaustion of HIV-specific CD8 T cell responses is a defining characteristic of HIV-1 infection, that is not fully restored on antiretroviral treatment (ART) (1–4). The lifelong treatment challenges, significant global health care burden of HIV-1 infection and associated comorbidities have resulted in renewed efforts to overcome this persistent immune dysfunction that prevents a functional cure (5). Enhanced antiviral immune function and optimal restoration of endogenous CD8 T cells are therefore required to complement immune based HIV-1 cure and treatment strategies.

Burgeoning evidence indicates that the immune status of CD8 T cells is delineated by its metabolic fitness state leading to different functional capacity and disease outcome (6). During an acute infection, naïve CD8 T cells undergo metabolic reprogramming, switching from quiescent mitochondrial oxidative phosphorylation (OXPHOS) to aerobic glycolysis to meet the bioenergetic demands of activated effector T cells (Teff) (7). A characteristic of T cell activation is the increased expression of *de novo* produced glucose transporters supporting the glycolytic pathway (6). Glucose transporter 1(Glut-1), the major glucose transporter in T lymphocytes, is upregulated following T cell receptor (TCR) stimulation for the uptake of exogenous glucose. Conversion to T cell memory, following peak effector differentiation and contraction, is characterized by a metabolic shift to mitochondrial OXPHOS and ability to utilize fatty acid oxidation (FAO) and increased spare respiratory capacity (SRC) (8, 9). Overall these changes facilitate stronger bursts of glycolysis and OXPHOS following re-stimulation. Mitochondrial activity has been shown to play a central role in the activation and maintenance of virus-specific responses (10, 11). Contrasting resolving viral infections, virus-specific CD8 T cell function is severely compromised in persistent infections (12).

During chronic HIV-1 infection under conditions of continuous activation T cell memory fails to develop efficiently leading to exhausted CD8 T cells (Tex). The process of exhaustion encompasses different T cell subsets within the spectrum of differentiation, including precursor exhausted (Tpex) and terminally exhausted subsets. Tpex CD8 T cells, expressing the transcription factor TCF-1, maintain stemness and undergo long-term self-renewal and are thought to differentiate into terminally exhausted populations (13). Tex are marked by progressive loss of effector function coupled to sustained upregulation of inhibitory receptors (IRs) and altered epigenetic, transcriptional and metabolic profiles (14). In animal models of chronic viral infections developing Tex cells display a number of metabolic derangements that includes suppressed cellular respiration and dysregulated mitochondrial energetics (15). A number of pathways underline these bioenergetic abnormalities, including PGC1a, a key transcriptional regulator of energy metabolism and mitochondrial biogenesis (16). Some of these metabolic changes have been observed in chronic viral infections such as HBV, where fundamental mitochondrial defects limit CD8 T cell antiviral responses (17, 18). More recently the metabolic plasticly of CD8 T cells has been associated with natural control of HIV-1 infection (19), whereas the loss of natural HIV-1 control has been linked to metabolic dysregulation (20). Notably glucose dependency was found to be a hallmark of dysfunctional HIV/SIV-specific CD8 T cells in HIV-1 positive subjects on suppressive ART and infected macaques with high viraemia (19). Along these lines, Glut-1 is found to be upregulated on CD4 T cells in HIV-1 infection and is associated with systemic immune activation and disease progression, irrespective of virological suppression with ART (21).

Early metabolic changes/dysregulation precede the emergence of exhaustion and are linked to inhibitory signals from the immunoregulatory receptor, Programmed cell death-1 (PD-1) (16, 22). However, Tex cells are known to be heterogeneous and exist at different states of exhaustion further defined by distinct transcriptional programmes (23). A range of exhausted states among CD8 T cells is associated with immune dysregulation (reflected by a low CD4/CD8 ratio) and response to ART (24). In HIV-1 infection, Tex skewed toward a T-bet^low^EOMES^hi^ expression profile characterize a terminally functionally exhausted state with higher levels of multiple co-inhibitory receptors (25). The CD8 T-bet^hi^EOMES^low^ T cell subset represents an intermediate exhausted population with proliferative potential (26). Upregulation and co-expression of multiple co-inhibitory molecules such as PD-1, T cell immunoreceptor with Ig and ITIM domains (TIGIT), TIM3 and LAG3 have been associated with T cell exhaustion (2, 27–29). Notably, these phenotypic signatures are linked to the time of HIV-1 viral rebound post standardized treatment interruption (30) and loss of viral control in the rare elite controllers (EC), who spontaneously control HIV-1 for a number of years in the absence of ART (31). The metabolic profile of CD8 Tex cells in HIV-1 infection remains, however, under-investigated. Bridging this knowledge gap could be critical in informing strategies to bolster T cell immunity.

To gain a better understanding of the metabolic factors that control the effector function and exhaustion of CD8 T cells in HIV-1 infection we examined the metabolic features of Tex populations in individuals with viraemic HIV-1 infection compared to individuals with natural control of HIV-1 infection (ECs) and HIV-1 seronegative controls. We also evaluated new approaches to restore underlying defects and stimulate recovery of HIV-specific effector function.

## Materials and Methods

### Study Subjects

The study included cryopreserved PBMCs from 14 treatment naïve patients with chronic HIV-1 infection (HIV-1) (Male= 11, Female= 3, median age= 46, range= 28-60, mean Log_10_VL=4.75, range= 3.08-6.42; median CD4 T cell count=310 cells/µL, range=11-720 cells/µL) and 10 elite controllers (ECs) (Male= 6, Female= 4, median age= 54, range= 41-63). ECs had at least two consecutive plasma HIV-1 RNA values <50 copies/mL for at least 12 months, and a median CD4 count=750 cells/µL, range= 545-1557, (32). Six HIV-1 positive individuals were followed up for at least 12 months after ART treatment initiation and had an undetectable HIV-1 RNA. Participants were recruited at Mortimer Market Centre for Sexual Health and HIV Research and the Royal Free Hospital (London, UK) following written informed consent as part of a study approved by the local ethics board committee. Fourteen demographically age, sex and lifestyle matched HIV-1 seronegative individuals were included for comparison, from whom blood was taken and cryopreserved for later use with written informed consent in accordance with the Declaration of Helsinki. All study participants were anti-Hepatitis C virus antibody negative and anti-HBsAg negative. Patient characteristics are included in **Supplementary Table 1** and **Supplementary Table 2**.

### Phenotypic and Metabolic Flow Cytometric Analysis

The following fluorochrome-conjugated antibodies were used in this study:

CD14 BV510, CD19 BV510, CD3 BV650, CD8 BV785, PD-1 BV711 or BV421, CD38 BV605 (BioLegend), TIGIT PE (eBioscience) for surface antigens; T-bet BV421 (BioLegend), EOMES PE-eFluor 610 (eBioscience) Glut-1 Alexa Fluor 647 (abcam), PGC-1 α Alexa Fluor 647 (Santa Cruz Biotechnology) for intracellular or intranuclear antigens. Briefly, Purified PBMCs were thawed and rested for an hour at 37°C in complete RPMI medium (RPMI 1640 supplemented (RPMI supplemented with Penicillin-Streptomycin, L-Glutamine, HEPES, non-essential amino acids, 2-Mercaptoethanol, and 10% Foetal bovine serum (FBS)). Cells were then washed, resuspended in PBS, and surface stained at 4°C for 20 min with saturating concentrations of different combinations of antibodies in the presence of fixable live/dead stain (Invitrogen). Cells were then fixed and permeabilized for detection of intracellular antigens. The Foxp3 intranuclear staining buffer kit (eBioscience) was used according to the manufacturer’s instructions for the detection of intranuclear markers. Assessment of mitochondrial mass, mitochondrial membrane potential and ROS production was performed by incubation with Mito-Tracker Green (100 nM) (Invitrogen), JC-1 (2 μM) or 5 μM MitoSOX Red (Invitrogen) respectively according to manufacturer’s instructions before surface staining. Samples were acquired on a BD Fortessa X20 using BD FACSDiva8.0 (BD Bioscience) and data analyzed using FlowJo 10 (TreeStar).

### CD3 Activation Functional Assay

For CD3 activation, 96-well flat-bottom plates (Nunc) were coated with 1 μg ml^–1^ with anti-human CD3 (clone OKT3, Biolegend) or an isotype-matched control antibody (mIgG1κ, BD Biosciences) overnight at 4°C. Plates were washed with sterile PBS before addition of 5 × 10^5^ PBMC per well. Cells were stimulated for 6 hours in the presence of 20 U ml^–1^ rhIL-2, GolgiStop (containing Monensin, 2 μmol/L), and GolgiPlug (containing brefeldin A, 10 μg ml^–1^) (BD Biosciences).

### Intracellular Cytokine Stimulation Functional Assay

Purified PBMCs were thawed and rested overnight at 37°C and 5% carbon dioxide in complete RPMI medium. After overnight rest, PBMCs were stimulated for 6 hours with 2μg/mL HIV-1 Gag pools or cytomegalovirus (CMV) pp65 (JPT Peptide Technologies, Berlin, Germany) or with 0·005% dimethyl sulphoxide (DMSO) as a negative control in the presence of αCD28/αCD49d co-stim antibodies (1 μg ml^–1^) GolgiStop (containing Monensin, 2 μmol/L), GolgiPlug (containing brefeldin A, 10 μg ml^–1^) (BD Biosciences) and anti-CD107a APC-H7 antibody (BD Biosciences). After stimulation, virus-specific CD8 T cells were identified by interferon γ (IFN-γ) and Tumour necrosis factor (TNF-α) production. Briefly, cells were surface stained and then fixed and permeabilized (CytoFix/CytoPerm; BD Biosciences) followed by intracellular cytokine staining with IFN-γ PE-Cy7 (BD Biosciences), TNF-α FITC (BioLegend). Samples were acquired on a BD Fortessa X20 using BD FACSDiva8.0 (BD Bioscience) and data analysed using FlowJo 10 (TreeStar).

### IL-15 and MT-Antioxidant Treatment

To assess virus-specific CD8 T cells responses after IL-15 and mitochondria antioxidant treatment, PBMCs were thawed and pre-incubated with IL-15 (100 ng ml^–1^), MitoTempo (10 μM; Sigma) + Mdivi-1 (10 μM, Sigma) + M1 (20 μM, Sigma), or dimethyl sulphoxide (DMSO) as control in cRPMI media overnight at 37°C and 5% carbon dioxide. After overnight pre-treatment, cells were then stimulated with 2μg/mL HIV-1 Gag pools or cytomegalovirus (CMV) pp65 for 6 hours.

### CD8 T Cell Isolation

CD8 T cells were enriched from PBMCs using a negative-selection magnetic bead kit (Miltenyi Biotec) as per the manufacturer’s instructions (>90% purity and viability).

### Extracellular Flux Assays

Oxygen consumption rate (OCR) and extracellular acidification rate (ECAR) using an XFp Analyzer as indicated by the manufacturer (Seahorse XF Technology, Agilent). CD8 T cells were isolated from PBMCs using a negative-selection magnetic bead kit (Miltenyi Biotec). Purified CD8 T cells were seeded at 3x10^5^ cells per well in Seahorse cell plates pre-coated with Cell-Tak (Corning). CD8 T cells were incubated for 45 min in a CO2-free incubator at 37°C before loading the plate in the Seahorse analyzer. The oxygen consumption rate (OCR) and extracellular acidification rate (ECAR) were measured in XF RPMI medium supplemented with 10 mM glucose, 1 mM pyruvate and 2 mM glutamine in response to oligomycin (1 μM), FCCP (1.5 μM), rotenone/antimycin A (0.5 μM) and 2-DG (50 mM) (Agilent technologies). Maximum respiration is the average OCR values post-FCCP injection. The spare respiratory capacity (SRC) was as the OCR values post-FCCP injection minus basal OCR. Glycolysis was calculated as the basal ECAR values minus post-2-DG injection values. Glycolytic reserve was calculated as post-rotenone/antimycin A injection ECAR values minus basal ECAR values.

### Unsupervized Analysis

To evaluate the co-expression of the markers at a single cell level and to identify various cell clusters with similar phenotypic profiles, unsupervized multidimensional analysis was performed using different algorithms in Cytobank (https://www.cytobank.org) (33). viSNE (visualization of high-dimensional single-cell data) uses the Barnes-Hut implementation (34). Cells were manually gated for lymphocytes, singlets, live cells, and CD3^+^CD8 and then subjected to viSNE analysis. viSNE clustering analysis was performed on 8 parameters (Glut-1, PD-1, TIGIT, EOMES, T-bet, CD38, CCR7, and CD45RA). Equal event sampling was selected across all samples. The FlowSOM algorithm, uses Self-Organizing Maps (SOMs) to define different clusters based on selected markers and reveal related phenotypic clusters. The samples were subjected to viSNE analysis before running FlowSOM with equal event sampling. The number of metaclusters was set to 7-10, number of the clusters was set to 100 and the size of clusters was set to relative with 15 pixels as Max relative size (Cytobank default).

### Statistics

Prism 8 (GraphPad Software) was used for statistical analysis as follows: the Mann–Whitney *U*-test was used for single comparisons of independent groups, Wilcoxon signed-rank test was used to compare two paired groups. The non-parametric Spearman test was used for correlation analysis. The statistical significances are indicated in the figures (*p <0.05, **p <0.01, ***p <0.001, and ****p <0.0001). Polyfunctionality tests were performed in SPICE version 6.0. PCA of participants flow cytometric parameters were conducted using the FactoMinR package in R Studio (Version 3.5.1). Flow measures were standardized prior to PCA. The values for PCA dimension 1 and 2 were extracted and plotted for each participant using ggplot.

## Results

### Glut-1 Upregulation Marks Exhausted CD8 T Cells in HIV-1 Infection

**Figure 1:**
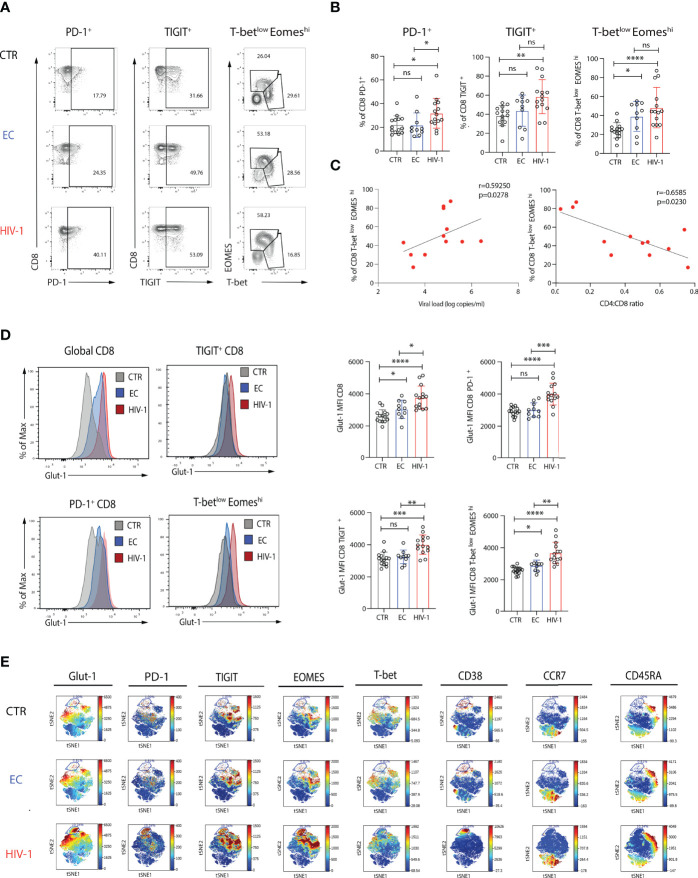
Increased expression of Glut-1 on exhausted CD8 T cells from viraemic HIV-1 infected patients. **(A)** Representative flow plots of percentage of PD-1^+^, TIGIT^+^, and T-bet^low^ EOMES^hi.^ in CD8 T cells from an HIV-1 negative control (CTR), an elite controller (EC), a viraemic HIV-1 positive donor (HIV-1) and **(B)** summary data from n = 14 HIV-1 negative controls (CTR) n = 10 elite controllers (ECs), n = 14 viraemic HIV-1 positive donors (HIV-1). **(C)** Correlation between percentage of T-bet^low^EOMES^hi^ CD8 T cells from n = 14 viraemic HIV-1 positive donors and viral load (log copies/ml) or CD4:CD8 ratio. **(D)** Representative histograms and summary data of Glut-1 expression on global CD8 T cells, PD-1^+,^ TIGIT^+^, and T-bet^low^ EOMES^hi^ CD8 T cells in n = 14 HIV-1 negative controls (CTR) n = 10 elite controllers (ECs), n = 14 viraemic HIV-1 positive donors (HIV-1). **(E)** ViSNE analysis of multiparametric flow data from concatenated files was performed on CD8 T cells from the CTR, EC, and HIV-1 positive individuals showing expression of Glut-1, PD-1, TIGIT, EOMES, T-bet, CD38, CCR7, and CD45RA. Each point on the viSNE map represents a single cell and color depicts intensity of protein expression. The non-parametric Spearman test was used for correlation analysis. Significance determined by Mann-Whitney *U* test, ns: non-significant, *p < 0.05, **p < 0.01 , ***p < 0.001, ****p < 0.0001.

To assess the capacity for glucose uptake by CD8 T cells in HIV-1 infection, including exhausted subpopulations, we analysed the expression of Glut-1 directly *ex-vivo.* Confirming previous observations, PD-1 and TIGIT expression levels were higher on CD8 T cells from viraemic HIV-1 individuals compared to ECs and HIV-1 seronegative controls (**Figures 1A, B**). A skewed balance towards a T-bet^low^EOMES^hi^ profile was more prominent in viraemic HIV-1 positive individuals and ECs in comparison to HIV-1 seronegative individuals (**Figures 1A, B**). The frequency of CD8 T-bet^low^EOMES^hi^ T cell population correlated positively with HIV-1 plasma viral load (pVL) and negatively with the CD4:CD8 ratio and percentage of CD4 T cells (**Figure 1C** and **Supplementary Figure 1A**). The levels of Glut-1 expression followed the pattern of expression of inhibitory receptors and T-bet^low^EOMES^hi^ profile on CD8 T cells from viraemic HIV-1 infected individuals relative to the control groups (**Figure 1D**).

To visually identify phenotypically distinguishable CD8 T cell subsets and global co-expression patterns in our study groups, we employed non-linear dimensionality reduction using t-SNE (35). A prominent cluster delineated by the expression of Glut-1^+^PD-1^hi^T-bet^low^EOMES^hi^TIGIT^+^CD38^+^CD45RA^-^CCR7^-^ was enriched in HIV-1 viraemic individuals in keeping with a terminally exhausted Teff population compared to ECs and HIV-1 negative subjects (**Figure 1E**). Terminally exhausted (Tex^term^) and intermediate exhausted CD8 T cells (Tex^int^), defined on the basis of differential combinations of surface inhibitory molecules (PD-1, TIGIT), transcription factors (T-bet, EOMES) and CD38 expression, and confirmed by manual gating, were more abundant in viraemic HIV-1 infected patients compared to the other groups (**Supplementary Figures 1B-F**). The frequency of Tex^term^ CD8 T cells (defined as PD-1^hi^T-bet^low^EOMES^hi^TIGIT^+^CD38^+^) correlated with the CD4 T cell count in HIV-1 viraemic patients (**Supplementary Figure 1G**). These populations were characterized by higher levels of expression of Glut-1 (**Supplementary Figure 1H**). However, whereas PD-1^-^ and Tex^int^ were responsive to TCR stimulation, reduced functional responses were observed in Tex^term^ cells (**Supplementary Figures 2A, B**). PD-1^-^ and Tex^int^ were able to further upregulate Glut-1 expression following TCR stimulation, but no difference in the levels of expression of Glut-1 were observed for Tex^term^ cells (**Supplementary Figures 2C, D**). These data suggest that Tex^term^ have already mobilized all available stores for Glut-1 expression and remain unable to increase their energy demands to stimulation reflected in their reduced functionality.

### Opposing Profiles in HIV-Specific Compared to CMV-Specific CD8 T Cells

**Figure 2:**
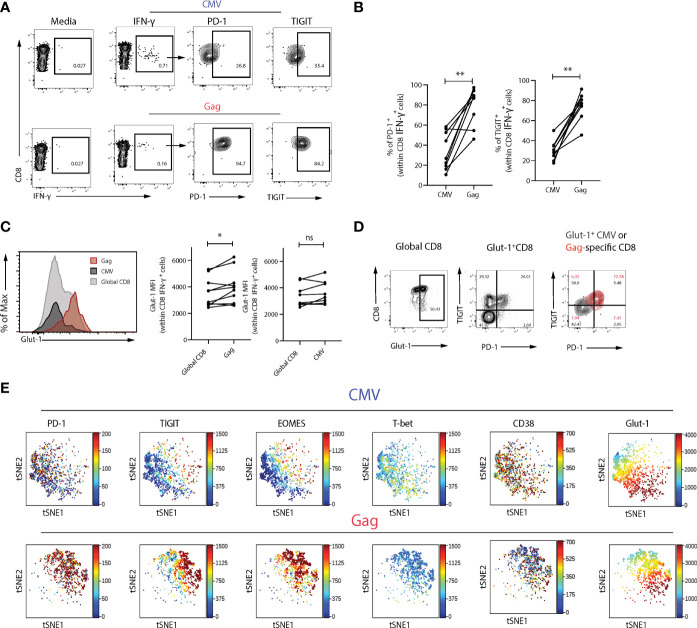
Glut-1 expression is enriched within exhausted Gag-specific compared to CMV-specific CD8 T cells. **(A)** Representative flow plots of PD-1 and TIGIT expression in Gag- and CMV-specific CD8 T cells following 6-hour stimulation with HIV-1 (Gag) or CMV (pp65) peptides directly *ex-vivo*. **(B)** Summary data of frequency of PD-1^+^ or TIGIT^+^ cells within Gag or CMV virus-specific populations from n = 9 viraemic HIV-1 infected donors in paired samples. **(C)** A representative histogram overlay of Glut-1 MFI in global, Gag- and CMV-specific CD8 T cells from n = 9 viraemic HIV-1 positive donors and summary data comparing Glut-1 MFI in global and Gag- or CMV-specific CD8 T cells in paired samples. **(D)** Flow plots of Glut-1 expression in global CD8 T cells and Gag and CMV-specific CD8 T cells (overlaid) co-expressing TIGIT and PD-1. **(E)** ViSNE analysis of Gag or CMV-specific CD8 T cells from n = 5 viraemic HIV-1 individuals showing expression of PD-1, TIGIT, EOMES, T-bet, CD38, and Glut-1. Wilcoxon matched-pairs signed rank test,*p < 0.05, **p < 0.005. ns, non-significant.

Progressive loss of function and exhaustion is a hallmark of HIV-specific CD8 T cells, whereas CMV-specific T cells differ in their phenotype and ability to respond following stimulation with their cognate peptide. We therefore examined the expression of Glut-1 in CD8 T cells directed against HIV-1 and CMV upon activation within the same viraemic HIV-1 infected individuals. Virus-specific populations were identified *via* IFN-γ staining, following stimulation with HIV-1 (Gag) or CMV (pp65) peptides directly *ex-vivo*. Expression of PD-1 and TIGIT were higher in HIV-specific CD8 T cells compared to CMV-specific CD8 T cells (**Figures 2A, B**). Glut-1 MFI was found to be significantly higher within HIV-specific CD8 T cells compared to global CD8 T cells in the same individual, whereas no difference was observed between global and CMV-specific CD8 T cells in Glut-1 MFI (**Figure 2C**). In line with higher expression of PD-1 and TIGIT in HIV-specific relative to CMV-specific CD8 T cells, we observed an enrichment of Glut-1 in HIV-specific CD8 T cells co-expressing PD-1 and TIGIT (**Figure 2D**). To extend the findings obtained *via* manual gating of flow cytometry data we visualized the profiles of HIV-specific and CMV-specific CD8 T cells on a viSNE map. Distinct topographical clusters were apparent depending on virus specificity with a predominant exhausted signature (PD-1^hi^EOMES^hi^T-bet^low^TIGIT^+^) co-expressing Glut-1 in HIV-specific CD8 T cells (**Figure 2E**). Consistent with lower PD-1 expression and more functional CD8 T cells in ECs, HIV-specific CD8 T cells from ECs exhibited comparable levels of Glut-1 expression to global CD8 T cells (**Supplementary Figures 3A, B**).

### Depressed Oxidative Phosphorylation in CD8 T Cells in Viraemic HIV-1 Infection

**Figure 3:**
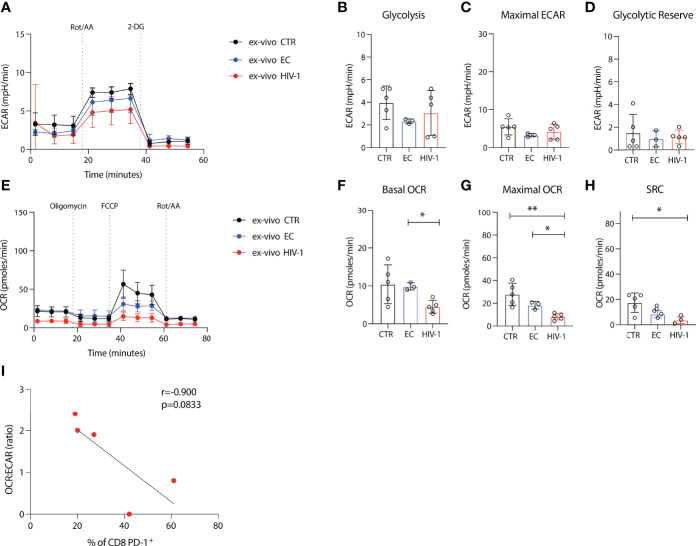
Depressed oxidative phosphorylation in CD8 T cells in viraemic HIV-1 infection. **(A)** Cellular extracellular acidification rate (ECAR) of purified CD8 T cells measured in real time *ex-vivo* **(B)** Glycolysis, **(C)** Maximal ECAR, and **(D)** glycolytic reserve in isolated CD8 T cells from n = 5 HIV-1 negative controls (CTR) n = 3 elite controllers (ECs), n = 5 viraemic HIV-1 positive donors (HIV-1). **(E)** Oxygen consumption rate (OCR) of purified CD8 T cells measured in real time *ex-vivo* **(F)** Basal OCR **(G)** maximal OCR and **(H)** Spare respiratory capacity (SRC) in purified CD8 T cells from n = 5 HIV-1 negative controls (CTR) n = 3 elite controllers (ECs), n = 5 viraemic HIV-1 positive donors (HIV-1). **(I)** Correlation between percentage of PD-1^+^ CD8 T cells from n = 5 viraemic HIV-1 positive donors and OCR : ECAR ratio. Sample duplicates were used for Seahorse assays. Significance determined by Mann-Whitney *U* test, *p < 0.05, **p < 0.01.

To probe further the metabolic profile and bioenergetic differences of the more exhausted CD8 T cells in viraemic HIV-1 subjects compared to ECs and HIV-1 negative controls we utilized seahorse technology. Glycolysis was assessed in purified CD8 T cells from each group by measurement of the cellular extracellular acidification rate (ECAR); a measure of lactate production and anaerobic glycolysis at the basal level and after rotenone and antimycin A addition, targeting complex I and complex III of the electron transport chain (ETC) respectively, and 2-deoxy-glucose (2-DG) a competitive inhibitor of glycolysis (**Figure 3A**). Basal glycolytic activity of CD8 T cells was similar between viraemic HIV-1 infected, ECs and HIV-1 negative donors (**Figure 3B**). No significant differences were detected in maximal glycolysis and the glycolytic reserve rate between the study groups (**Figures 3C, D**).

Next, we determined the oxygen consumption rates (OCR), a measure of oxidative phosphorylation (OXPHOS) and a key metric of mitochondrial function, under basal conditions and following the addition of a stressor mix including oligomycin (an ATP synthesis inhibitor), carbonyl cyanide-4 (trifluoromethoxy) phenylhydrazone (FCCP; uncoupling synthesis of ATP from the ETC), and rotenone and antimycin A (**Figure 3E**). Lower basal OCR was observed in viraemic HIV-1 positive patients with decreased maximal OCR in CD8 T cells from viraemic individuals compared to healthy donors and ECs (**Figures 3E–G**). HIV-1 donors had lower SRC relative to HIV-1 negative control individuals (**Figure 3H**).

To examine the relevance of exhaustion as a driver of the observed metabolic changes we correlated the expression of PD-1 on CD8 T cells from our viraemic donors with the OCR : ECAR ratio. Despite the low numbers a trend towards an inverse correlation was observed suggestive of a cellular preference/dependence on glycolysis and decreased oxygen consumption of PD-1 expressing CD8 T cells in viraemic HIV-1 infection (r= -0.9, p=0.0833) (**Figure 3I**).

### Exhausted CD8 T Cells Are Associated With Mitochondrial Defects

**Figure 4:**
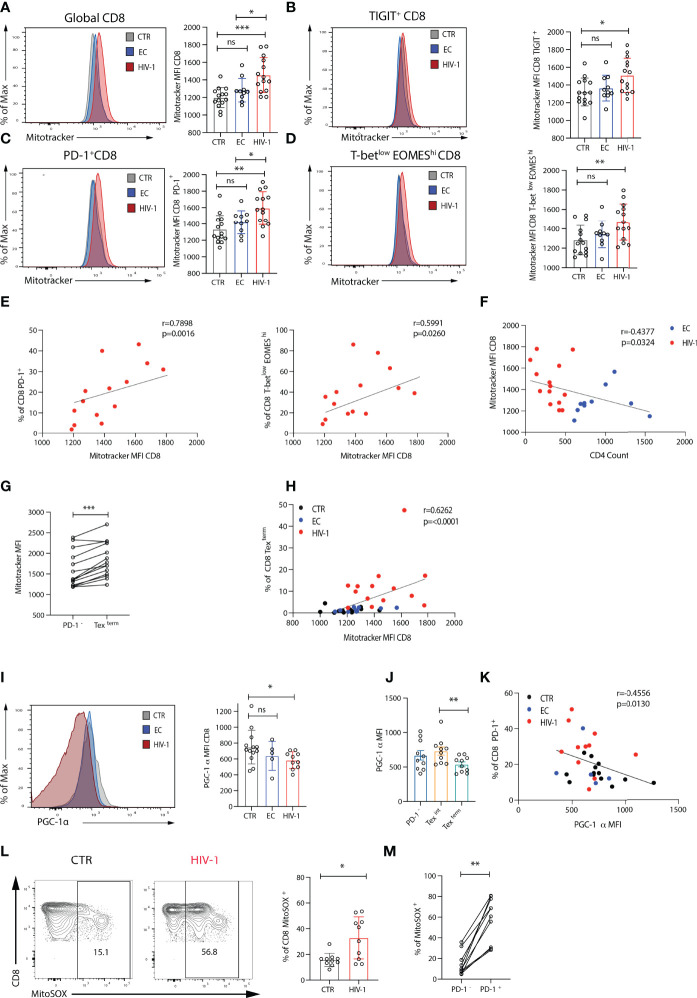
Exhausted CD8 T cells from viraemic HIV-1 positive individuals are associated with mitochondrial impairment. **(A)** Comparison of mitochondria mass in global, **(B)** TIGIT^+^, **(C)** PD-1^+^, **(D)** T-bet^low^EOMES^hi^ CD8 T cells from n = 14 HIV-1 negative controls (CTR) n = 10 elite controllers (ECs), n = 14 viraemic HIV-1 positive individuals by Mitotracker Green staining, representative histograms, and summary data. **(E)** Correlation between percentage of PD-1^+^ and T-bet^low^EOMES^hi^ CD8 T cells and Mitotracker green (Mitotrtacker) MFI in CD8 T cells from n = 14 viraemic HIV-1 positive donors. **(F)** Correlation between Mitotracker MFI in CD8 T cells and CD4 count in EC and HIV-1 positive donors. **(G)** Comparison of Mitotracker MFI in PD-1^-^ and terminally exhausted (Tex^term^) (T-bet^low^EOMES^hi^PD-1^+^TIGIT^+^CD38^+^) CD8 T cells in paired samples from viraemic HIV-1 positive patients. **(H)** Correlation between percentage of terminally exhausted (Tex^term^) and Mitotracker MFI in CD8 T cells from the study groups. **(I)** A representative histogram of PGC-1α expression in CD8 T cells from an HIV-1 negative control (CTR), an elite controller (EC), and a viraemic HIV-1 positive donor (HIV-1), and summary data from n = 12 HIV-1 negative controls (CTR) n = 5 elite controllers (ECs), n = 10 viraemic HIV-1 positive donors (HIV-1). **(J)** Comparison of PGC-1α expression in PD-1^-^, intermediate (Tex^int^) and terminally exhausted (Tex^term^) CD8 T cells in paired samples from viraemic HIV-1 positive patients. **(K)** Correlation between percentage of PD-1^+^ CD8 T cells and PGC-1α expression in CD8 T cells from the study group. **(L)** A representative histogram of the MitoSOX staining in CD8 T cells from an HIV-1 negative control (CTR), and a viraemic HIV-1 positive donor (HIV-1), and summary data from n = 10 HIV-1 seronegative controls (CTR) and n = 10 viraemic HIV-1 positive donors (HIV-1). **(M)** Comparison of percentage of MitoSOX^+^ cells in PD-1^-^ and PD-1^+^ CD8 T cells in paired samples from viraemic HIV-1 positive patients. The non-parametric Spearman test was used for correlation analysis. Bars show mean ± SD. **p < 0.01, ***p < 0.001. ns, non-significant, *p < 0.05.

The decrease in maximal respiration and SRC in CD8 T cells from viraemic HIV-1 donors suggested a reduced ability of these cells to produce energy engaging OXPHOS in response to stress or stimulation. To investigate whether they had a mitochondrial defect, we initially assessed mitochondrial mass (MM), *via* staining cells with the mitochondrial-potential independent dye MitoTracker green, that has been previously utilized to evaluate the presence of enlarged dysfunctional mitochondria in T cells (36). PD-1, TIGIT and EOMES^hi^T-bet^low^ expressing CD8 T cells from viraemic HIV-1 infected donors had a higher MM *ex-vivo* compared to ECs and HIV-1 negative donors (**Figures 4A–D**), with PD-1^+^ and EOMES^hi^T-bet^low^ CD8 T cells correlating positively with MitoTracker green MFI (r=0.7789, p=0.0016; r=05991, p=0.0260 respectively) (**Figure 4E**). An inverse correlation between MM and CD4 T cell count was noted (r=0.4377, p=0.0324) (**Figure 4F**). To further explore mitochondrial fitness, we assessed the mitochondria membrane potential by using the ratiometric fluorescent dye JC-1. We observed a trend towards more depolarised mitochondria in global CD8 T cells in HIV-1 infection as evidenced by the lower red/green fluorescence intensity ratio (**Supplementary Figures 4A, B**). This trend was more evident in Tex (T-bet^low^EOMES^hi^PD-1^+^) CD8 T cells (**Supplementary Figures 4C, D**).

Next, we used principal components analysis (PCA) to evaluate the relationship between immune cell profiles, Glut-1 and MM in the study groups. Eleven measures were included in the PCA. Derived principal component 1 and 2 explained 44.2% and 17.0% of the variation in the data, respectively. HIV-1 viraemic patients were separated from HIV-1 negative donors in PCA space, whereas EC overlapped between viraemic HIV-1 positive and HIV-1 negative subjects (**Supplementary Figure 5A**). Participant values in HIV-1 positive group for principal component 1 were significantly higher compared to EC and HIV-1 negative donors (**Supplementary Figure 5B**). The PD-1^hi^T-bet^low^EOMES^hi^TIGIT^+^CD38^+^CD45RA^-^CCR7^-^ CD8 T cell cluster is enriched for MitoTracker green (**Supplementary Figure 5C**), consistent with the patterns of Glut-1 expression. Higher MM level was observed in CD8 Tex^term^ cells compared with PD-1^-^ CD8 T cells (**Figure 4G** and **Supplementary Figures 5D, E**), demonstrating a strong positive association with Mitotracker green MFI (**Figure 4H**).

These findings suggested that dysregulated mitochondria within exhausted CD8 T cells are not able to maintain/support their bioenergetic demands during established chronic HIV-1 infection. This prompted us to measure the expression of peroxisome proliferator-activated receptor γ (PPARγ) coactivator 1α (PGC-1α); a key transcriptional regulator of energy metabolism genes and mitochondrial biogenesis (37). PGC-1α expression was reduced in global CD8 T cells from viraemic HIV-1 donors compared to ECs and HIV-1 negative participants particularly within Tex^term^ populations (**Figures 4I–J**). Notably there was an inverse correlation between the levels of expression of PD-1 and PGC-1α expression in CD8 T cells (r=-0.4556, p=0.0130) (**Figure 4K**) suggesting that PD-1 could influence metabolic programmes through PGC-1α repression in exhausted CD8 T cells.

T cell activation and effector function does not only rely on mitochondrial biogenesis but also balanced production of reactive oxygen species (ROS) (11, 38). Given the more pronounced differences in mitochondria between viraemic HIV-1 infected individuals and HIV-1 seronegative controls we evaluated next the levels of ROS directly *ex-vivo* in these two groups. Significantly higher levels of mitochondrial superoxide (MitoSOX), a measure of ROS, were detected *ex-vivo* in CD8 T cells from patients with chronic viraemic HIV-1 infection compared with cells from healthy individuals and MitoSOX was enriched within PD-1^+^ expressing cells (**Figures 4L, M**). ROS staining was confirmed in healthy individuals *ex-vivo* and following Rotenone treatment, known to induce ROS production through a blockade of electron transfer in complex I, limiting oxidative phosphorylation (39) (**Supplementary Figures 6A, B**). Overall, the inability of exhausted CD8 T cells in viraemic HIV-1 infection to utilize OXPHOS could be partly due to mitochondrial alterations/reconfiguration and changes in ROS production (40).

### Functional Restoration of Exhausted HIV-Specific CD8 T Cells by Mitochondrial Targeted Treatment in Combination With IL-15

**Figure 5:**
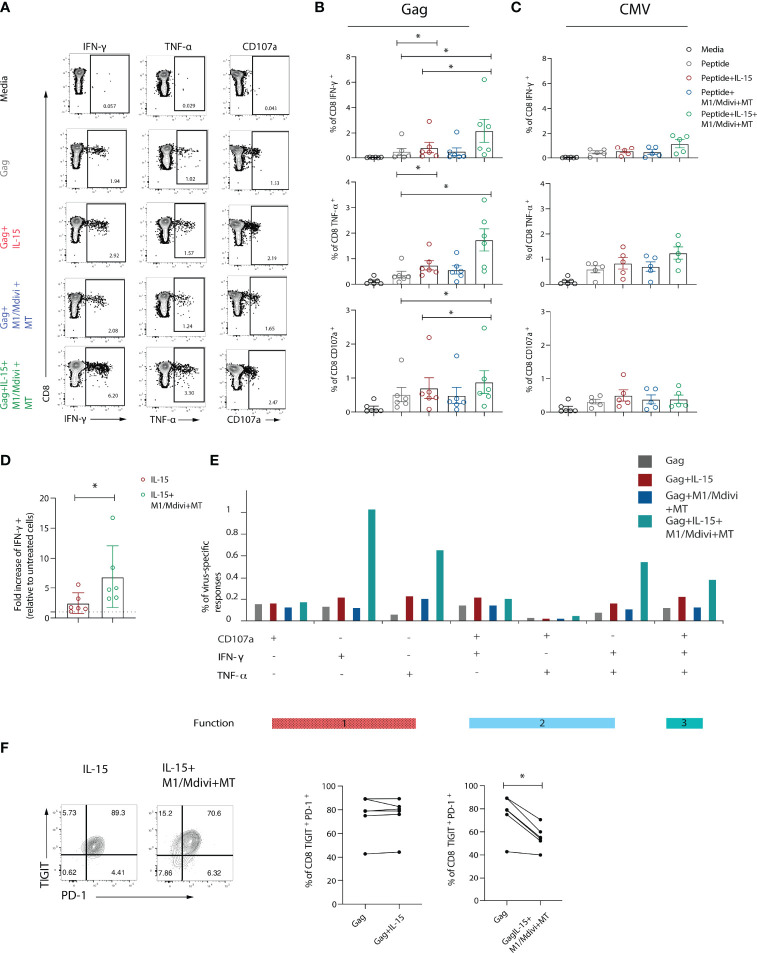
Targeting mitochondrial dysfunction in combination with IL-15 improves functional capacity of exhausted HIV-specific CD8 T cells. **(A)** Representative flow plots and **(B)** Summary data showing percentage of IFN-γ, TNF-α and CD107a positive CD8 T cells after 6-hour stimulation with Gag peptides in the presence or absence of IL-15, and M1, mdivi and MitoTempo (M1/Mdivi+MT). **(C)** Summary data showing the percentage of IFN-γ, TNF-α and CD107a positive CD8 T cells after 6-hour stimulation with CMV (pp65) peptides in the presence or absence of IL-15, and M1, mdivi and MitoTempo (M1/Mdivi+MT). **(D)** Fold increase of the level of IFN-γ response to Gag peptide stimulation upon treatment with IL-15 alone (IL-15) or combined with M1, mdivi and MitoTempo (IL-15+M1/Mdivi+MT). **(E)** SPICE bar charts of IFN-γ, TNF-α and CD107a responses in Gag-specific CD8 T cells showing 1 function, 2 functions, or 3 functions of following treatment with different conditions. **(F)** Representative flow plots and summary data of percentage of TIGIT^+^PD-1^+^ co-expression in Gag-specific CD8 T cells in the presence of IL-15 (IL-15) alone or combined with M1, mdivi and MitoTempo (M1/Mdivi+MT). Wilcoxon matched-pairs signed rank test, *p < 0.05.

Taking into consideration these mitochondrial alterations and compromised OXPHOS, which are required to fuel HIV-specific CD8 T cell responses in addition to glycolysis (19), we reasoned that restoring mitochondrial function could improve CD8 T cell antiviral responses. We therefore focused our efforts on targeting mitochondria through treatment with the antioxidant piperidine-nitroxide MitoTempo (MT), a mitochondria-specific superoxide scavenger (18, 41), and the small-molecule inhibitors of mitochondrial fission, mdivi-1 and fusion promoter (M1), to pharmacologically enhance mitochondrial fusion and increase respiratory capacity (42). This approach was used alone or in combination with IL-15, a cytokine that promotes mitochondrial biogenesis in CD8 T cells, in order to assess their ability to improve antiviral responses. To that end, we pre-treated PBMCs form patients with chronic viraemic HIV-1 infection with IL-15 alone and/or M1/Mdivi+MT overnight prior to short-term peptide stimulation with HIV-1 Gag or CMV-pp65 (**Figure 5A**). Although IL-15 pre-treatment increased cytokine production following Gag peptide stimulation, an even greater enhancement was observed for IFN-γ, TNF-α, and CD107a production when IL-15 was combined with M1/Mdivi+MT (**Figure 5B**). By contrast no significant changes were observed for CMV-specific CD8 T cell responses following IL-15 alone or combination treatment in keeping with their opposing and more functional profiles compared to exhausted HIV-specific CD8 T cells (**Figure 5C**). Pre-treatment of IL-15 combined with M1/Mdivi+MT resulted in a higher fold change of IFN-γ production compared with IL-15 pre-treated cells, following Gag stimulation (**Figure 5D**). Double-positive IFN-γ/TNF-α and polyfunctional CD8 T cell responses associated with an enhanced control of HIV-1 infection (43) were increased following combination treatment (**Figure 5E**). IL-15+M1/Mdivi+MT treatment led to decreased frequency of PD-1^+^ TIGIT^+^ cells within HIV-specific CD8 T cells, suggesting that such an approach can potentially shift T cells towards a less exhausted phenotype with increased function (**Figure 5F**).

### Persistent Metabolic Exhaustion of CD8 T Cells During Suppressive ART Amenable to Reversal

**Figure 6:**
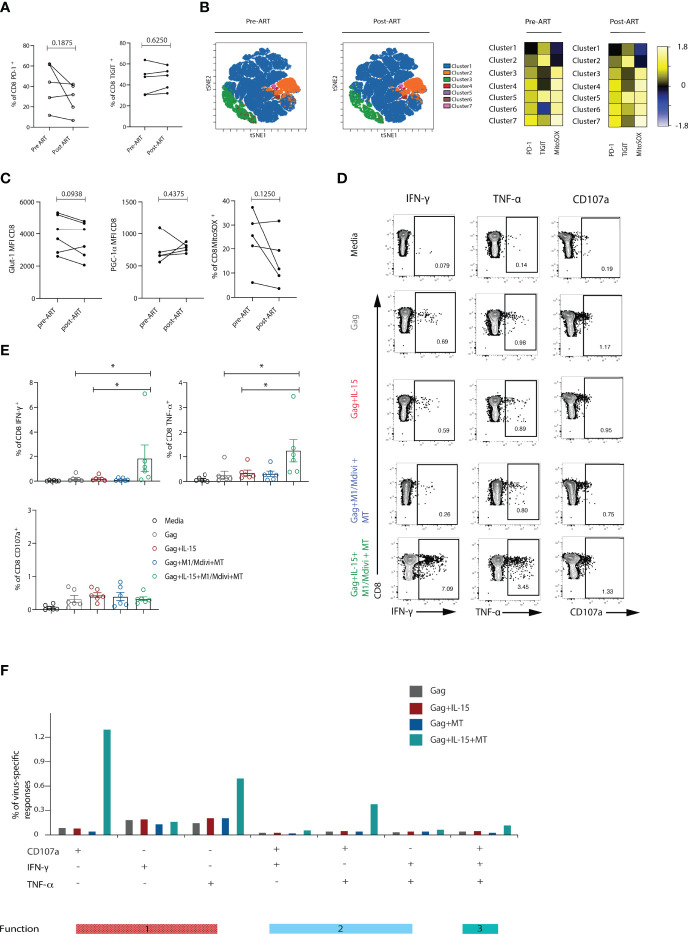
Metabolic abnormalities persisted in ART-suppressed patients. **(A)** Longitudinal analysis of the frequency of PD-1^+^ or TIGIT^+^ CD8 T cells from HIV-1 positive individuals before and after 12 months of ART suppression (n = 5). **(B)** viSNE map of FlowSOM clustering of CD8 T cells in longitudinal samples. Heatmap showing expression of TIGIT and PD-1 markers for each cluster; Representative flow plots and **(C)** Glut-1 expression (n = 6), PGC-1α expression (n = 5) and percentage of MitoSOX^+^ (n = 5) CD8 T cells from HIV-1 positive individuals before and after ART treatment. **(D)** Representative flow plots and **(E)** Summary data showing percentage of IFN-γ, TNF-α and CD107a positive CD8 T cells after 6-hour stimulation with Gag peptides in the presence or absence of IL-15, and M1, mdivi and MitoTempo (MT) in n = 6 HIV-1 ART treated individuals. **(F)** SPICE analysis bar charts of IFN-γ, TNF-α and CD107a responses in Gag-specific CD8 T cells showing 1 function, 2 functions, or 3 functions of Gag-specific CD8 T cells following treatment with different conditions. Wilcoxon matched-pairs signed rank test, *p < 0.05.

Functional cure interventions are aimed at ART stabilized HIV-positive patients and ongoing CD8 T cell dysfunction could therefore hinder such attempts. To evaluate the degree of CD8 T cell reconstitution we performed a longitudinal assessment of CD8 T cells in a sub-group of HIV-1 positive individuals before and 12 months after the introduction of suppressive ART. Paired analysis of the data showed a persistent signature of exhaustion in global CD8 T cells characterized by higher expression of multiple inhibitory receptors (**Figure 6A**). The stability of this phenotype is further evident by analysis with self-organizing maps (FlowSOM) as a clustering tool to enable visualization of metaclusters based on phenotypic similarities (**Figure 6B**). This analysis did not show any striking differences between the groups. Notably no significant changes were noted in the expression of Glut-1, PGC-1α and levels of ROS in keeping with ongoing metabolic abnormalities (**Figure 6C**). Accordingly, we found that a combination of IL-15+ M1/Mdivi+MT pre-treatment increased the frequency of HIV-specific CD8 T cell responses from individuals on ART (**Figures 6D, E**), and had no effect on the frequency of CMV-specific responses in the same donors (data not shown), in keeping with findings in viraemic infection. Combination treatment increased both IFN-γ, TNF-α and double-positive IFN-γ/TNF-α and polyfunctional CD8 T cell responses following Gag peptide stimulation (**Figure 6F**). These results show that metabolic reprogramming *via* mitochondial targeting led to enhanced HIV-specific responses in treated individuals that could help enhance T cell fitness to induce HIV-1 remission.

## Discussion

With increased appreciation of the importance of immunometabolism in shaping effective CD8 T cell responses this study provides new insights into the metabolic features of exhausted CD8 T cells in HIV-1 infection and identifies potential new targets for therapeutic restoration of antiviral responses. The emerging picture from this study highlights the spectrum of exhaustion states in CD8 T cells and virus-specific populations directed against HIV-1 and identifies cellular perturbations centered on mitochondrial dysfunction. The latter likely impacts on a number of energy requiring processes leading to defective antiviral T cell effector function (11) that can be partially recovered *via* mitochondrial targeting and IL-15 treatment.

Our findings show that a more terminally exhausted CD8 T cell profile, on the basis of expression PD-1^hi^EOMES^hi^T-bet^low^TIGIT^+^ and upregulation of Glut-1, characterizes viraemic infection that persists despite effective ART and differentiates HIV-specific from the more functional CMV-specific CD8 T cells. Individuals with natural control of HIV-1 infection (ECs), have an intermediate phenotype in relation to HIV-1 negative controls and HIV-1 viraemic individuals that is not accompanied by impaired mitochondrial OXPHOS and mitochondrial defects. These data reinforce recent observations of upregulated genes associated with activation, exhaustion and glycolysis in HIV-1 non-controllers, whereas HIV-1 controllers exhibited greater metabolic plasticity and preserved mitochondrial metabolism linked to their functional advantage (19). Along these lines tight glucose dependency has been associated with poor HIV/SIV specific CD8 T cell responses in blood and tissue compartments, inflicting limitations on CD8 T cell function *in vivo* as shown in the tumour microenvironment (44).

Although a defect in glucose uptake cannot be formally excluded without appropriate validated glucose transport assays in T cells (45), no differences in maximal glycolysis and glycolytic reserve rate were observed in CD8 T cells between the study groups. Instead, decreased oxidative phosphorylation in CD8 T cells from chronically infected HIV-1 donors indicated a potential mitochondrial defect. A number of previous studies have indicated that mitochondrial energetics, homeostasis and plasticity are key determinants of T cell fate (7, 16, 42). In this study, the presence of increased mitochondrial mass correlated with the terminal exhaustion status of CD8 T cells in viraemic HIV-1 infection. Increased mitochondrial mass has been previously linked to a survival defect in HIV-specific CD8 T cells (46). These findings are reminiscent of observations in HBV infection, where exhausted HBV-specific CD8 T cells demonstrate an inability to switch from glycolysis to oxidative phosphorylation, accompanied by an increase in mitochondrial size (17) and extensive mitochondrial gene dysregulation (18). Mitochondria have also been reported to be the main targets of PD-1 inhibitory signalling affecting cristae morphology in human CD8 T cells (47) in addition to influencing metabolic programmes in exhausted CD8 T cells (16, 22). Along these lines, repression of PGC-1α, a central transcriptional regulator of oxidative metabolism and mitochondrial biogenesis, was prominent in terminally exhausted CD8 T cells in viraemic HIV-1 infection. These defects were accompanied by elevated ROS production and notably persisted despite effective virological suppression with ART in longitudinal analysis. Together these data support a prominent role of mitochondrial dysfunction in the setting of chronic viral infection associated CD8 T cell exhaustion. Relevant to this, exhausted CD8 T cells have been reported to display a marked downregulation of genes encoding specific mitochondrial proteins (18). These include carnitine palmitoyl transferase 1a (CPT-1a), which is involved in the utilization of fatty acids as an alternative energy source and T cell memory formation (9), and in keeping with recent observations of lower fatty acid uptake in CD8 T cells from HIV-1 non-controllers and a lower fatty acid:glucose ratio (19). Of note the delicate balance between mitochondrial fusion and fission has also been described to influence respiratory capacity and quality control of mitochondria in CD8 T cells (42), with the mitochondrial fusion protein, optic atrophy protein 1(OPA-1) being downregulated in exhausted T cells (48). Although dysregulated mitochondrial homeostasis may be a common feature of exhausted T cells in chonric viral infections and cancer 16–18, 49–51, there may be some important distinctions relating to a specific viral protein or the local tissue microenvironment which will be of interest to delineate in future studies.

Given the underlying mitochondrial defects in HIV-1 infection, we posited that a broader approach capable of targeting multiple pathways would be required to improve mitochondrial function/metabolism in exhausted CD8 T cells which are enriched within HIV-specific CD8 T cells. To this end we found that a combination approach with a ROS scavenger, pharmacological inhibitors of fission to encourage fusion and increase SRC, and IL-15 led to increased frequency and functionality of HIV-specific CD8 T cells. This effect was selectively observed in HIV-specific T cells versus the less exhausted CMV-specific T cells within the same individuals that presumably engage mitochondrial respiration to sustain their activity. IL-15 is known to promote FAO and mitochondrial biogenesis in CD8 T cells and enhance the survival and function of HIV-specific CD8 T cells (19). Accordingly, IL-15 pre-treatment alone was able to afford a degree of functional restoration in HIV-specific CD8 T cells from viraemic patients. However, this effect was further enhanced in combination with mitochondrial targeted treatments reinforcing the importance of a multipronged approach to simultaneously recover multiple defects that persist despite ART and improve HIV-specific T cell functionality. In this study we have not evaluated the effects of individual treatments on mitochondrial phenotypes, which will need to be addressed further in larger HIV-1 cohorts.

IL-15 or IL-15 superagonists have already entered clinical trials showing promising results in animal and preclinical models and currently being evaluated as a strategy to clear HIV-1 reservoirs (NCT02191098). Reducing superoxide content by Mito-tempo, has been also shown to lead to enhanced antiviral CD8 T cell responses to HBV (18). Our results point to the potential of combining IL-15 treatment with compounds that manipulate mitochondria to improve cellular energetics and have implications for the development of therapeutic strategies aimed at reinvigorating Tex in chronic viral infections. Future studies are warranted focusing on the interconnection between exhaustion, nutrient availability and mitochondrial alterations impacting T cell function in HIV-1 infection that could help identify additional therapeutic targets that can be safely manipulated to increase CD8 T cell metabolic fitness in HIV-1 infection.

## Data Availability Statement

The raw data supporting the conclusions of this article will be made available by the authors, without undue reservation.

## Ethics Statement

Participants were recruited at Mortimer Market Centre for Sexual Health and HIV Research and the Royal Free Hospital (London, UK) following written informed consent as part of a study approved by the local ethics board committee. The patients/participants provided their written informed consent to participate in this study.

## Author Contributions

AA, EM-C performed experiments, acquisition of data, analysis and drafting of the manuscript. DH-B contributed to data analysis. RM, AS, SR-J contributed to study design, data interpretation and critical editing of the manuscript. DP contributed to conception and design of study, data analysis and interpretation, critical revision of the manuscript and study supervision. All authors contributed to the article and approved the submitted version.

## Funding

This work was supported by MRC grant MR/M008614 and NIH R01AI55182 (DP). AA is supported by a Saudi Ministry of Education studentship. DH-B is supported by the Wellcome Trust Genomic Medicine and Statistics DTP (award number 108861/B/15/Z).

## Conflict of Interest

The authors declare that the research was conducted in the absence of any commercial or financial relationships that could be construed as a potential conflict of interest.

## Publisher’s Note

All claims expressed in this article are solely those of the authors and do not necessarily represent those of their affiliated organizations, or those of the publisher, the editors and the reviewers. Any product that may be evaluated in this article, or claim that may be made by its manufacturer, is not guaranteed or endorsed by the publisher.

## Supplementary Material

Supplementary Table 1 Cohort characteristics.

Supplementary Table 2 Cohort characteristics of longitudinal analysis of HIV-1 positive individuals before and after 12 months of ART suppression.

Supplementary Figure 1 Chronic HIV-1 infection elicits heterogenous subsets of exhausted CD8 T cells in HIV-1 non-controllers. **(A)** Correlation between the frequency of T-bet^low^ EOMES^hi^ CD8 T cells and the frequency of CD4 T cells. **(B)** viSNE map of FlowSOM clustering of concatenated files was performed on CD8 T cells from n=14 HIV-1 negative controls (CTR) n=10 elite controllers (EC), n=14 viraemic HIV-1 positive donors (HIV-1). **(C)** abundance of the selected cluster in total CD8 T cells (multiplied by 1000). **(D)** Distribution of intensities of selected markers is shown in a histogram for: PD-1, TIGIT, EOMES, T-bet, CD38, and, Glut-1. Bar plots showing the abundance of each major cluster. **(E)** Representative flow plots showing gating strategy to define PD-1^-^ (CD8 PD-1^-^), intermediate exhausted (T-bet^hi^EOMES^low^PD-1^int^) terminally exhausted (T-bet^low^EOMES^hi^PD-1^+^TIGIT^+^CD38^+^) subsets in a viraemic HIV-1 positive donor. **(F)** Summary data of frequency of each population of total CD8 T cells from n=14 HIV-1 negative controls (CTR) n=10 elite controllers (ECs), and n=14 viraemic HIV-1 positive donors (HIV-1). **(G)** Correlation between the frequency of Tex^term^ CD8 T cells and CD4 T cell count in the HIV-1 and ECs groups. **(H)** Glut-1 expression on manually gated cell subsets: PD-1^-^, intermediate exhausted (Tex^int^), and terminally exhausted (Tex^term^) from n=14 viraemic HIV-1 positive donors, paired analysis.

Supplementary Figure 2 Tex^term^ cells failed to upregulate Glut-1 expression following 18-hour TCR stimulation. **(A)** Representative flow plot and **(B)** summary data showing percentage of TNF-α and TNFα+IFN-γ+ CD8 T cells from n=8 viraemic HIV-1 donors after 6-hour stimulation with 1 μg ml^–1^ with anti-human CD3 in the presence of 20 U ml^–1^ rhIL-2. **(C)** Representative histograms and paired analysis data of Glut-1 expression on global CD8 T cells following 6- or 18-hour stimulation with anti-CD3 and IL-2 in n=6 HIV-1 negative controls (CTR) n=9 elite controllers (ECs), and n=8 viraemic HIV-1 positive donors (HIV-1). **(D)** Representative histograms and summary data of Glut-1 expression on intermediate exhausted (Tex^int^), and terminally exhausted (Tex^term^) from n=8 viraemic HIV-1 positive donors, paired samples.

Supplementary Figure 3 Similar level of Glut-1 expression was detected in Gag-specific compared to CMV-specific CD8 T cells from HIV-1 Elite controllers. **(A)** Representative flow plots and summary data of percentage of PD-1^+^ or PD-1^+^TIGIT^+^ double-positive cells within Gag-specific CD8 populations from n=5 elite controllers (ECs) and n=10 viraemic HIV-1 positive donors (HIV-1) **(B)** A representative histogram overlay of Glut-1 MFI in global, Gag- and CMV-specific CD8 T cells from n=5 ECs and summary data comparing Glut-1 MFI in global and Gag- or CMV-specific CD8 T cells in paired samples.

Supplementary Figure 4 Assessment of mitochondrial membrane potential. **(A)** Representative flow plots and **(B)** summary data of JC-1 red/green ratio in n=4 HIV-1 negative and HIV-1 viraemic donors. Mitochondria polarization and depolarization state is indicated by red and green fluorescence, respectively. **(C)** A representative flow plot of JC-1 red/green expression in global and overlaid T-bet^low^EOMES^hi^PD-1^+^CD8 T cells (shown in green). **(D)** Paired data of the JC-1 red/green ratio in global CD8 T cells and T-bet^low^EOMES^hi^PD-1^+^CD8 T cells from n=4 HIV-1 positive individuals.

Supplementary Figure 5 Analysis of mitochondrial mass in exhausted subsets. **(A)** PCA analysis of aggregated flow cytometric parameters and correlation between measures and PCA component 1 and 2. **(B)** Participants’ values for the principal component 1 in the study groups. **(C)** viSNE analysis of multiparametric flow data from concatenated files was performed on CD8 T cells from the CTR, EC, and HIV-1 positive groups showing marker intensity of Mitotracker, PD-1, TIGIT, EOMES, T-bet, CD38, CCR7, and CD45RA. Each point on the viSNE map represents a single cell and color depicts intensity of protein expression. **(D)** Distribution of intensity of Mitotracker is shown in a histogram form. **(E)** Comparison of Mitotracker MFI in PD-1^-^, intermediate (Tex^int^) and terminally exhausted (Tex^term^) CD8 T cells in paired samples from n=10 viraemic HIV-1 positive patients. Significance determined by Mann-Whitney *U* test, ns: non-significant, *p<0.05, **p<0.01.

Supplementary Figure 6 Analysis of ROS production following rotenone treatment. **(A)** Representative FACS plots of MitoSOX staining at basal level or following rotenone treatment (as positive control). **(B)** The percentage of MitoSOX^+^ CD8 T cells in paired samples from n=3 HIV-1 negative controls.
