# Supplementary material for: Functional Restoration of Exhausted CD8 T Cells in Chronic HIV-1 Infection by Targeting Mitochondrial Dysfunction
Source: Front Immunol. 2022 Jul 5;13:908697. doi: 10.3389/fimmu.2022.908697 (PMC9295450; doi:10.3389/fimmu.2022.908697)
Supplement: Supplementary Table 1 — Cohort characteristics. [file DataSheet_1.pdf]

Supplementary Table 1: Cohort characteristics.

Group	Age	Gender (Female:Male)	CD4 count cells/µL	Log_{10} Viral load
HIV-1 negative (CTR)	Median: 41 (26-49)	1:8	N/A	N/A
HIV-1 positive elite controllers (ECs)	Median: 49 (41-63)	4:7	Median: 750 (545-1557)	<50
Viraemic HIV-1 positive (HIV-1)	Median: 46 (28-60)	3:14	Median: 310 (11-720)	Mean: 4.82 (3.36-6.42)

Supplementary Table 2: Cohort characteristics of longitudinal analysis of HIV-1 positive individuals before and after 12 months of ART suppression.

PID	Age	Gender	CD4:CD8 (pre-ART)	CD4:CD8 (post-ART)	CD4 count cells/µL (pre-ART)	CD4 count cells/µL (post-ART)	Viral load (pre-ART)	Viral load (post-ART)	HIV-1 treatment regimen
1	46	M	1.89	2.55	690	879	10000	<50	Triumeq
2	37	M	0.65	1.17	310	270	8710	<50	Triumeq
3	49	M	0.88	1.16	720	1090	46000	<50	Truvada/Raltegravir
4	48	M	0.74	0.71	370	660	87096	<50	Truvada/Efavirenz
5	30	M	0.43	0.85	420	470	66068	<50	Truvada/Raltegravir
6	32	M	0.54	0.63	300	410	2300	<50	Kivexa/Raltegravir
