# Supplementary material for: Functional Restoration of Exhausted CD8 T Cells in Chronic HIV-1 Infection by Targeting Mitochondrial Dysfunction
Source: Front Immunol. 2022 Jul 5;13:908697. doi: 10.3389/fimmu.2022.908697 (PMC9295450; doi:10.3389/fimmu.2022.908697)

Supplementary figure 1

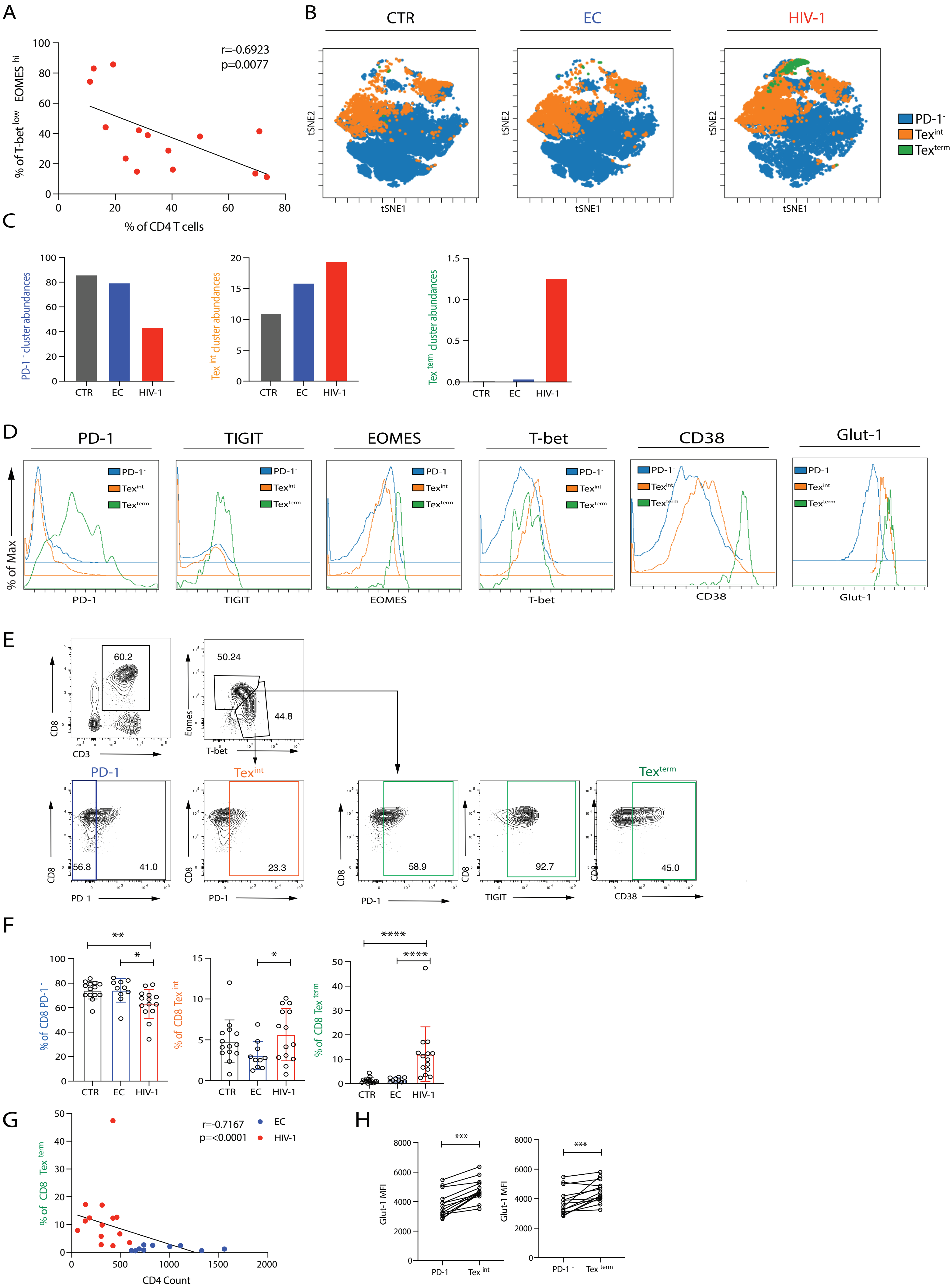

Supplementary figure 2

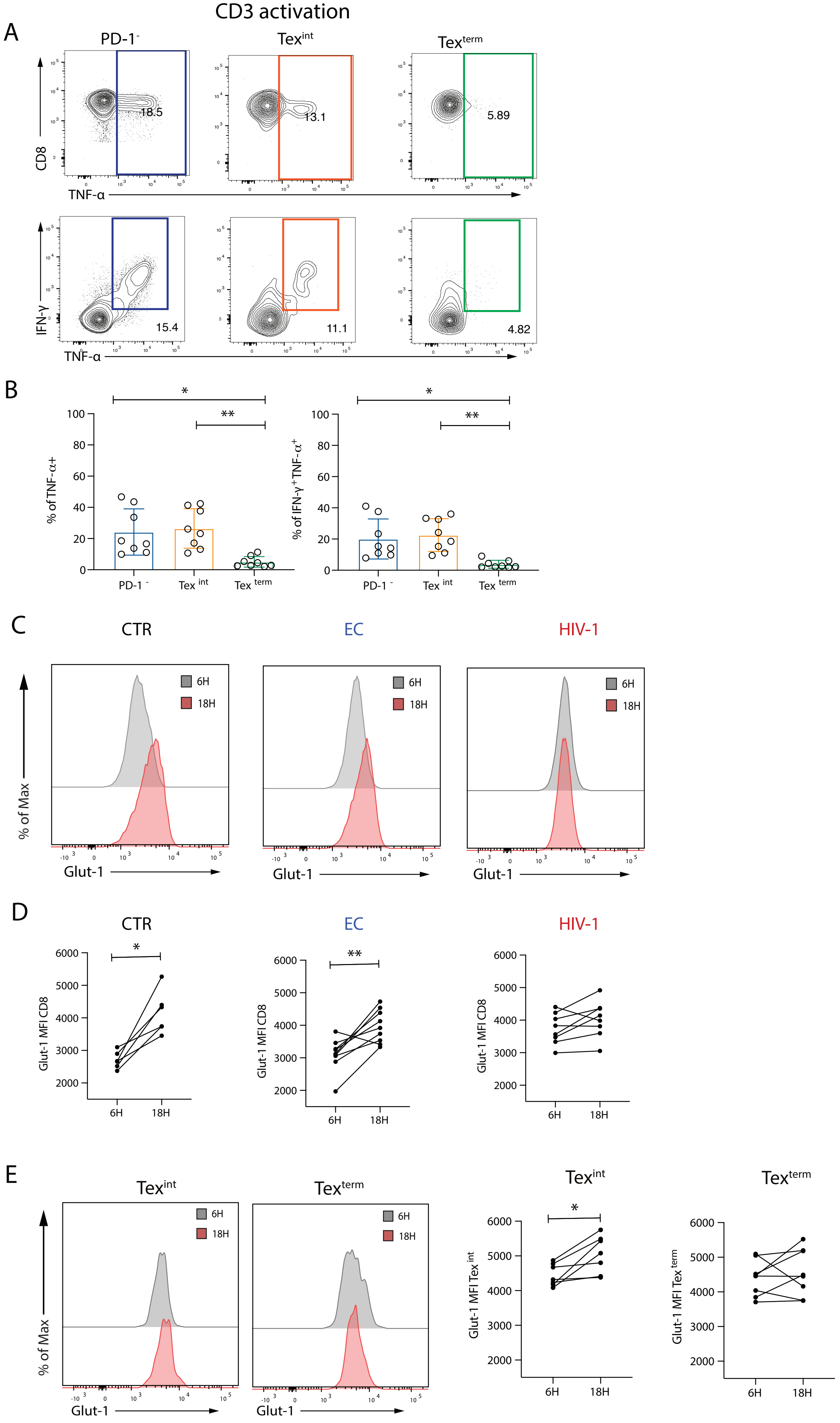

Supplementary figure 3

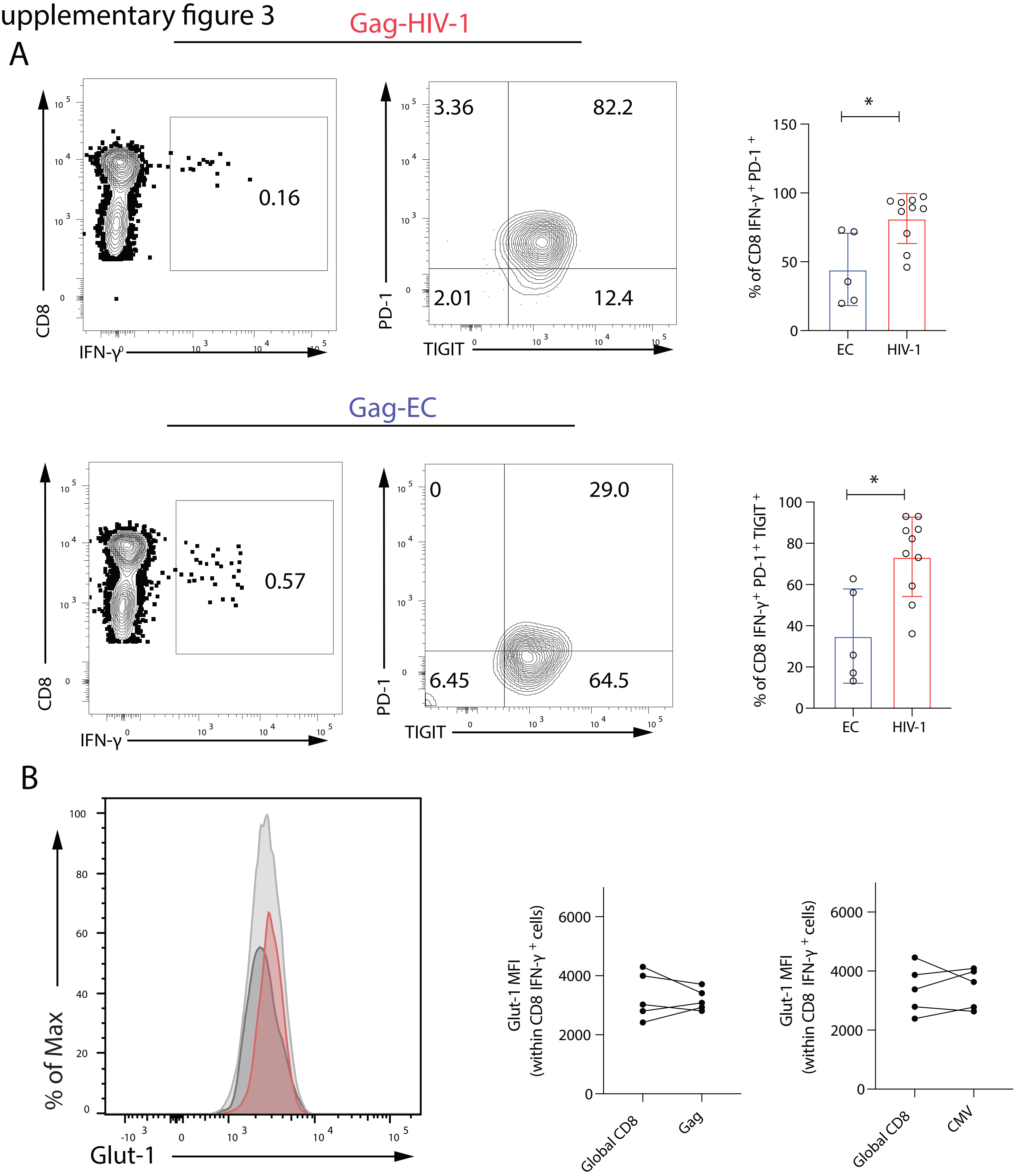

Supplementary figure 4

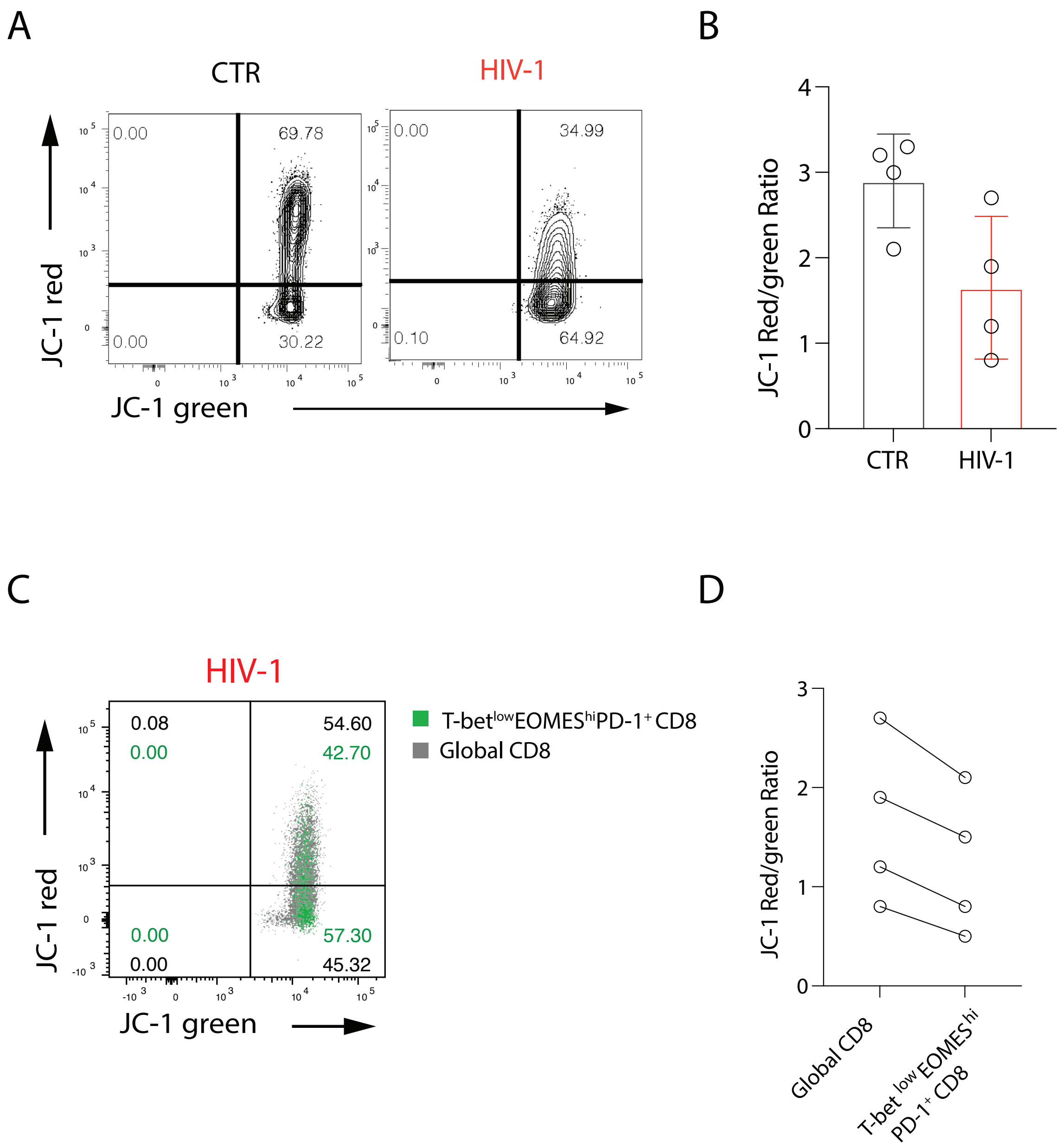

Supplementary figure 5

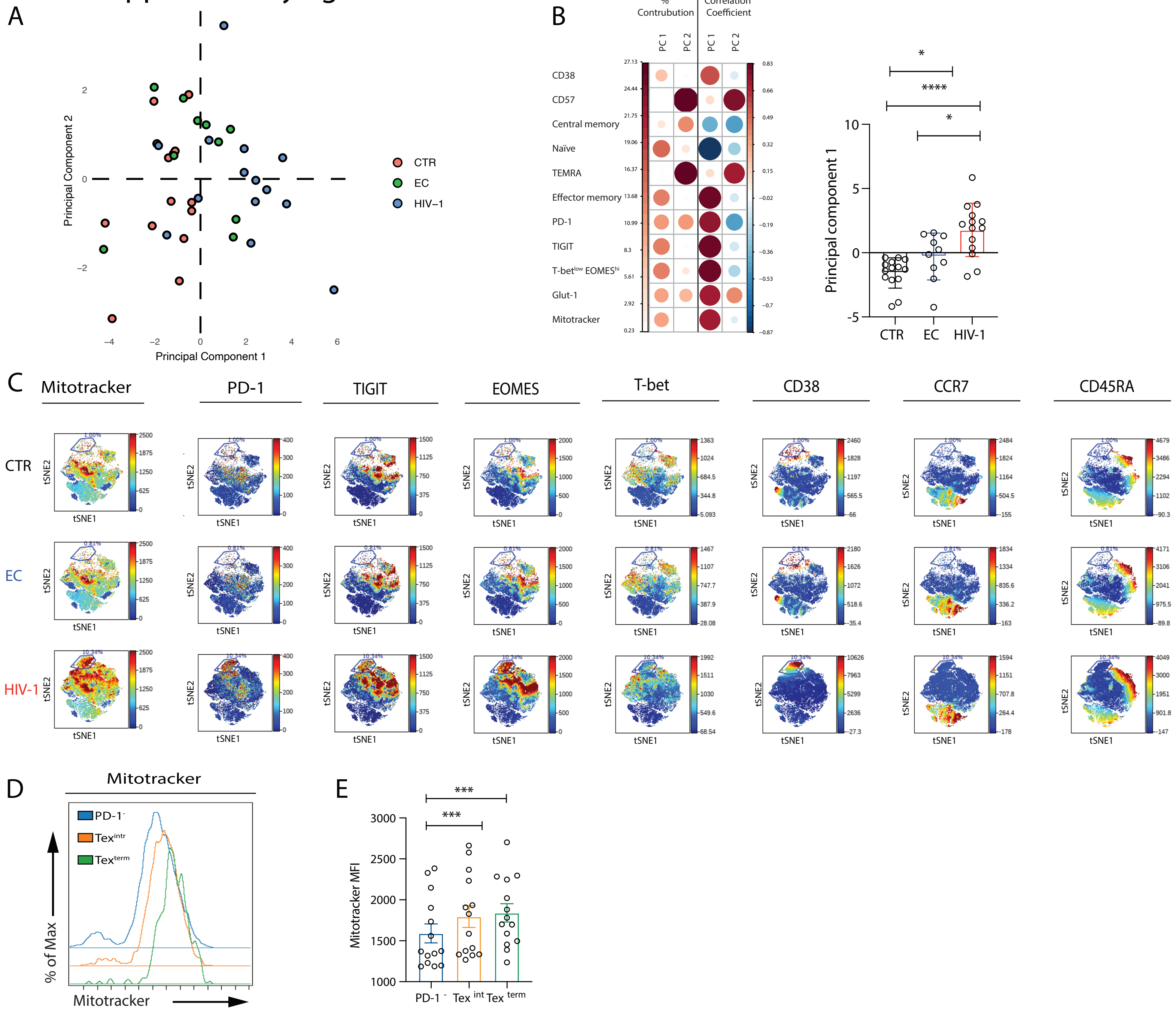

Supplementary figure 6

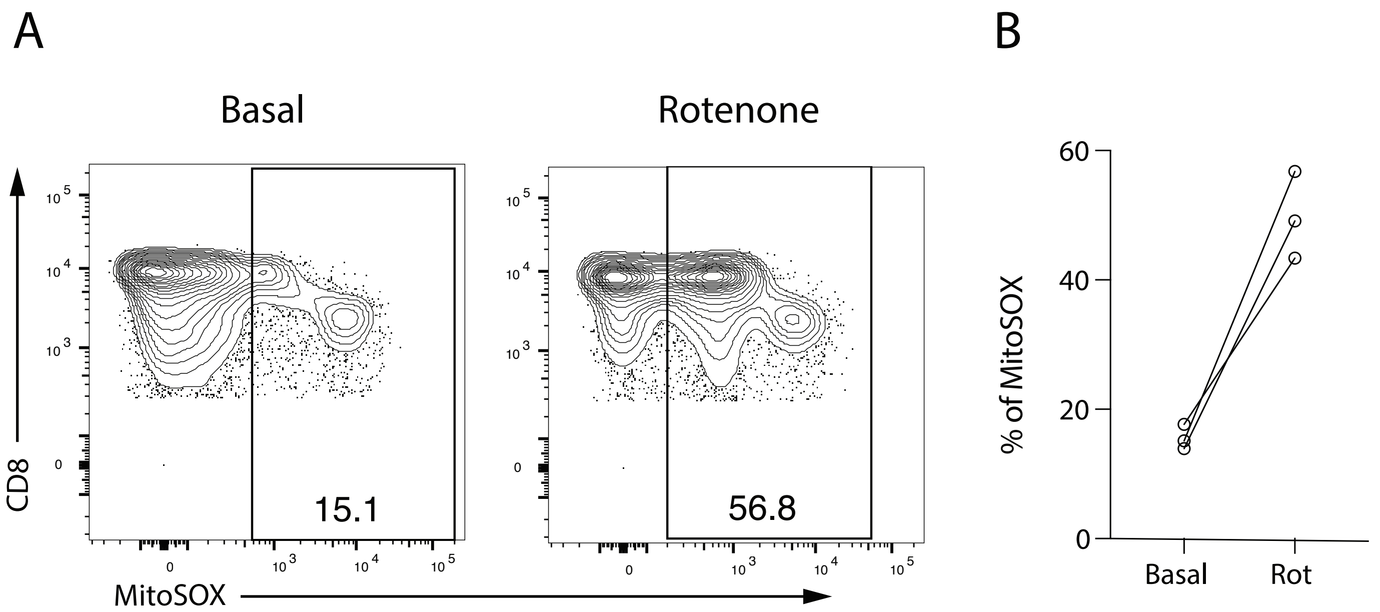

Supplement: Supplementary Figure 1 — Chronic HIV-1 infection elicits heterogenous subsets of exhausted CD8 T cells in HIV-1 non-controllers. (A) Correlation between the frequency of T-betlow EOMEShi CD8 T cells and the frequency of CD4 T cells. (B) viSNE map of FlowSOM clustering of concatenated files was performed on CD8 T cells from n=14 HIV-1 negative controls (CTR) n=10 elite controllers (EC), n=14 viraemic HIV-1 positive donors (HIV-1). (C) abundance of the selected cluster in total CD8 T cells (multiplied by 1000). (D) Distribution of intensities of selected markers is shown in a histogram for: PD-1, TIGIT, EOMES, T-bet, CD38, and, Glut-1. Bar plots showing the abundance of each major cluster. (E) Representative flow plots showing gating strategy to define PD-1- (CD8 PD-1-), intermediate exhausted (T-bethiEOMESlowPD-1int) terminally exhausted (T-betlowEOMEShiPD-1+TIGIT+CD38+) subsets in a viraemic HIV-1 positive donor. (F) Summary data of frequency of each population of total CD8 T cells from n=14 HIV-1 negative controls (CTR) n=10 elite controllers (ECs), and n=14 viraemic HIV-1 positive donors (HIV-1). (G) Correlation between the frequency of Texterm CD8 T cells and CD4 T cell count in the HIV-1 and ECs groups. (H) Glut-1 expression on manually gated cell subsets: PD-1-, intermediate exhausted (Texint), and terminally exhausted (Texterm) from n=14 viraemic HIV-1 positive donors, paired analysis. [file DataSheet_2.pdf]
